# Supplementary material for: Effects of Frequency Filtering on Intensity and Noise in Accelerometer-Based Physical Activity Measurements
Source: Sensors (Basel). 2019 May 11;19(9):2186. doi: 10.3390/s19092186 (PMC6539652; doi:10.3390/s19092186)
Supplement: Supplementary file 1 [file sensors-19-02186-s001.pdf]

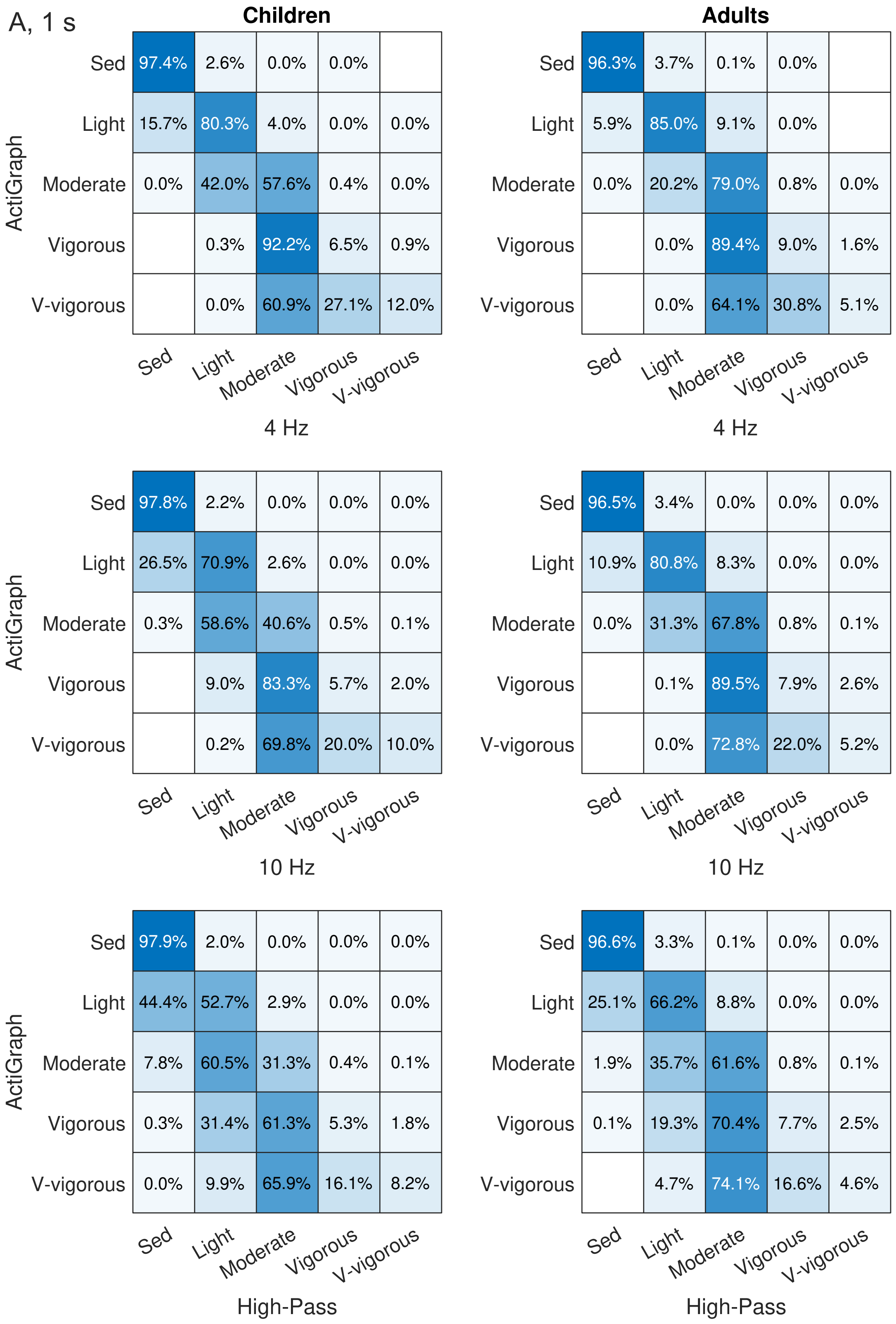
A, 1 s
Children
Adults
ActiGraph
Sed
Light
Moderate
Vigorous
V-vigorous
4 Hz
Children 4 Hz: Sed 97.4% 2.6% 0.0% 0.0%
Light 15.7% 80.3% 4.0% 0.0% 0.0%
Moderate 0.0% 42.0% 57.6% 0.4% 0.0%
Vigorous 0.3% 92.2% 6.5% 0.9%
V-vigorous 0.0% 60.9% 27.1% 12.0%
Adults 4 Hz: Sed 96.3% 3.7% 0.1% 0.0%
Light 5.9% 85.0% 9.1% 0.0%
Moderate 0.0% 20.2% 79.0% 0.8% 0.0%
Vigorous 0.0% 89.4% 9.0% 1.6%
V-vigorous 0.0% 64.1% 30.8% 5.1%
10 Hz
Children 10 Hz: Sed 97.8% 2.2% 0.0% 0.0% 0.0%
Light 26.5% 70.9% 2.6% 0.0% 0.0%
Moderate 0.3% 58.6% 40.6% 0.5% 0.1%
Vigorous 9.0% 83.3% 5.7% 2.0%
V-vigorous 0.2% 69.8% 20.0% 10.0%
Adults 10 Hz: Sed 96.5% 3.4% 0.0% 0.0% 0.0%
Light 10.9% 80.8% 8.3% 0.0% 0.0%
Moderate 0.0% 31.3% 67.8% 0.8% 0.1%
Vigorous 0.1% 89.5% 7.9% 2.6%
V-vigorous 0.0% 72.8% 22.0% 5.2%
High-Pass
Children High-Pass: Sed 97.9% 2.0% 0.0% 0.0% 0.0%
Light 44.4% 52.7% 2.9% 0.0% 0.0%
Moderate 7.8% 60.5% 31.3% 0.4% 0.1%
Vigorous 0.3% 31.4% 61.3% 5.3% 1.8%
V-vigorous 0.0% 9.9% 65.9% 16.1% 8.2%
Adults High-Pass: Sed 96.6% 3.3% 0.1% 0.0% 0.0%
Light 25.1% 66.2% 8.8% 0.0% 0.0%
Moderate 1.9% 35.7% 61.6% 0.8% 0.1%
Vigorous 0.1% 19.3% 70.4% 7.7% 2.5%
V-vigorous 4.7% 74.1% 16.6% 4.6%

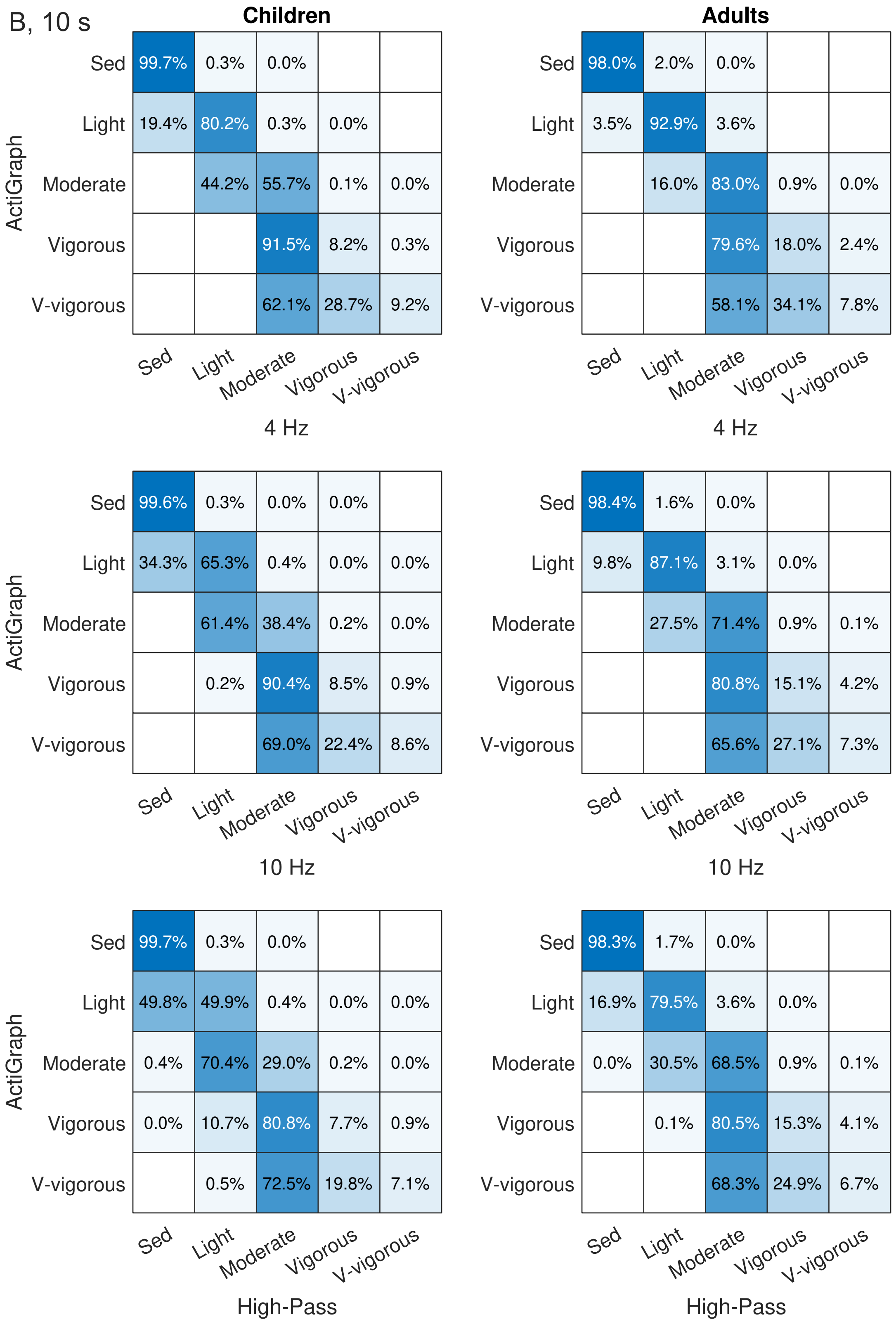
B, 10 s
Children
Adults
ActiGraph
Sed
Light
Moderate
Vigorous
V-vigorous
99.7% 0.3% 0.0%
19.4% 80.2% 0.3% 0.0%
44.2% 55.7% 0.1% 0.0%
91.5% 8.2% 0.3%
62.1% 28.7% 9.2%
4 Hz
98.0% 2.0% 0.0%
3.5% 92.9% 3.6%
16.0% 83.0% 0.9% 0.0%
79.6% 18.0% 2.4%
58.1% 34.1% 7.8%
4 Hz
99.6% 0.3% 0.0% 0.0%
34.3% 65.3% 0.4% 0.0% 0.0%
61.4% 38.4% 0.2% 0.0%
0.2% 90.4% 8.5% 0.9%
69.0% 22.4% 8.6%
10 Hz
98.4% 1.6% 0.0%
9.8% 87.1% 3.1% 0.0%
27.5% 71.4% 0.9% 0.1%
80.8% 15.1% 4.2%
65.6% 27.1% 7.3%
10 Hz
99.7% 0.3% 0.0%
49.8% 49.9% 0.4% 0.0% 0.0%
0.4% 70.4% 29.0% 0.2% 0.0%
0.0% 10.7% 80.8% 7.7% 0.9%
0.5% 72.5% 19.8% 7.1%
High-Pass
98.3% 1.7% 0.0%
16.9% 79.5% 3.6% 0.0%
0.0% 30.5% 68.5% 0.9% 0.1%
0.1% 80.5% 15.3% 4.1%
68.3% 24.9% 6.7%
High-Pass

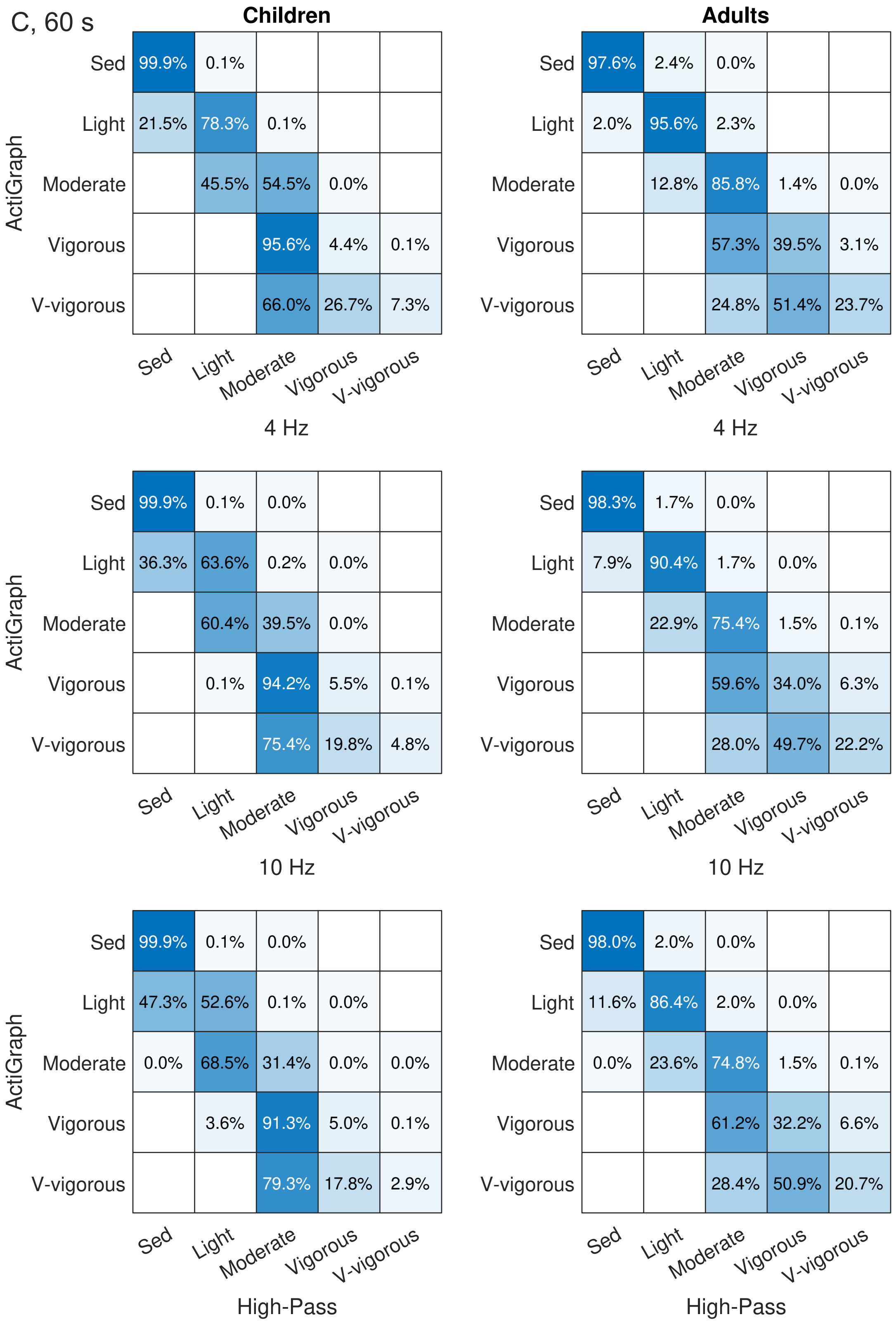
C, 60 s
Children
Adults
ActiGraph
Sed
Light
Moderate
Vigorous
V-vigorous
99.9%
0.1%
21.5%
78.3%
0.1%
45.5%
54.5%
0.0%
95.6%
4.4%
0.1%
66.0%
26.7%
7.3%
4 Hz
97.6%
2.4%
0.0%
2.0%
95.6%
2.3%
12.8%
85.8%
1.4%
0.0%
57.3%
39.5%
3.1%
24.8%
51.4%
23.7%
99.9%
0.1%
0.0%
36.3%
63.6%
0.2%
0.0%
60.4%
39.5%
0.0%
0.1%
94.2%
5.5%
0.1%
75.4%
19.8%
4.8%
10 Hz
98.3%
1.7%
0.0%
7.9%
90.4%
1.7%
0.0%
22.9%
75.4%
1.5%
0.1%
59.6%
34.0%
6.3%
28.0%
49.7%
22.2%
99.9%
0.1%
0.0%
47.3%
52.6%
0.1%
0.0%
0.0%
68.5%
31.4%
0.0%
0.0%
3.6%
91.3%
5.0%
0.1%
79.3%
17.8%
2.9%
High-Pass
98.0%
2.0%
0.0%
11.6%
86.4%
2.0%
0.0%
0.0%
23.6%
74.8%
1.5%
0.1%
61.2%
32.2%
6.6%
28.4%
50.9%
20.7%
